# Clinical characteristics, treatment and outcomes of adnexal torsion in pregnant women: a retrospective study

**DOI:** 10.1186/s12884-020-03173-7

**Published:** 2020-08-24

**Authors:** Yong-xue Wang, Shan Deng

**Affiliations:** Department of Obstetrics and Gynecology, Peking Union Medical College Hospital, Chinese Academy of Medical Science and Peking Union Medical College, Beijing, China

**Keywords:** Adnexal torsion, Conservative surgery, Emergency surgery, Laparoscopy; outcome, Pregnancy

## Abstract

**Background:**

Adnexal torsion during pregnancy is a gynecological emergency. Delayed diagnosis and treatment can cause ovarian necrosis and fetal loss. This study assessed the clinical characteristics, treatment and outcomes of adnexal torsion in pregnant women.

**Methods:**

A retrospective study was conducted at a tertiary center between January 2008 and January 2018. Eighty-two pregnant women with surgically confirmed adnexal torsion were included. The clinical characteristics, ultrasound data, surgical interventions and pregnancy outcomes were analyzed.

**Results:**

The median age of the patients was 28 (range, 18–38) years. The median gestational age was 11 (range, 6–31) weeks: 53 (64.6%) were in the first trimester, 21 (25.6%) were in the second trimester, and 8 (9.8%) were in the third trimester. The most common symptoms and signs were sudden pelvic pain (100%) and adnexal or pelvic masses (97.6%), followed by nausea and vomiting (61%). The Doppler blood flow signal disappeared in 62.5% of the patients. Sixty-three (76.8%) patients underwent laparoscopy, and 29 (24.2%) underwent laparotomy. The median gestational age in patients undergoing laparotomy was higher than that in those undergoing laparoscopy (26 weeks vs 10 weeks, *p* < 0.001). Fifty-three (64.6%) patients underwent conservative surgery, with 48 detorsions and cystectomies, 2 detorsions and cyst fenestrations, 1 detorsion only and 2 salpingectomies only. Twenty-nine (25.4%) patients underwent unilateral salpingo-oophorectomy. There were no cases of postoperative thrombosis, spontaneous abortion or recurrence during the same pregnancy. Seven patients underwent simultaneous artificial abortion. One patient experienced intrauterine fetal death, and 74 patients had live births.

**Conclusion:**

Surgical intervention was required as soon as possible. Laparoscopic conservative surgery is safe and may be appropriate to preserve ovarian function.

## Background

Adnexal torsion (AT), a true gynecological emergency, is the fifth most common cause of acute pelvic pain in women of reproductive age [1]. It refers to the complete or partial rotation of the adnexa around the ligamentous supports, which contain the vascular pedicle. After AT, the interruption of venous reflux and arterial flow could lead to ischemia of the adnexa and eventually to necrosis, resulting in permanent loss of the affected ovary. Pregnancy and assistant reproductive techniques have been reported to be risk factors for AT [2–4]. Although the incidence of AT during pregnancy is uncertain, estimates range from 0.2 to 3% [5–7].

AT can occur at any time during gestation, although it occurs more frequently during the first and early second trimesters [8]. However, the main symptoms are nonspecific, and it is easy to misdiagnose AT as other acute abdominal diseases, such as acute appendicitis, urinary obstruction, and ectopic pregnancy. Missed diagnosis of AT could lead not only to ovarian necrosis and sepsis but also threaten the pregnancy. Laparoscopy has been proven to be a safe approach for the treatment of AT in nonpregnant women [8]. Recent reports have indicated that conservative surgery, i.e., detorsion with or without cystectomy, does not increase thromboembolic complications and favors ovarian function preservation [9, 10].

However, the incidence of AT during pregnancy is very low. The pregnancy status and enlarged uterus may affect the treatment decision. There have been relatively few studies on AT during pregnancy, and only case reports or small series have been reported [3, 11–13]. Clear guidelines for the management of AT in pregnancy are lacking. This study was designed to report our 10 years of experience in a relatively large population of pregnant women with AT and to improve emergency diagnosis and treatment.

## Methods

A retrospective chart review was performed at Peking Union Medical College Hospital, a tertiary hospital in Beijing, China. Patients with a surgically confirmed diagnosis of AT during pregnancy from January 2008 to January 2018 were identified. Women with a diagnosis of AT who did not have torsion confirmed surgically were not included in this study. This retrospective chart review was approved by the ethics committee of Peking Union Medical College Hospital.

Clinical, ultrasound, surgical and pathological data were retrospectively retrieved from the medical records. The clinical information included the patient’s age, parity, mode of conception, gestational age, symptoms and signs, and white blood cell count. The following surgical information was recorded: time from onset to surgery, surgical approach, surgical procedure, and postoperative complications. Pregnancy outcomes included live birth, gestational week of delivery, and newborn weight. The pregnancy outcome of patients who continued their pregnancy follow-up and delivered in our hospital was obtained from their medical records. A telephone interview was performed only for patients who delivered at other hospitals. Ultrasound scans were performed by sonographers in the emergency department. Surgery was performed by attending physicians in the gynecology department.

The surgical procedure was classified as conservative surgery or unilateral salpingo-oophorectomy (USO). Conservative surgery included detorsion with cystectomy, detorsion with cyst fenestration, detorsion only and salpingectomy only. The gestational trimesters were classified as follows: (1) first trimester, 12 weeks or before; (2) second trimester, 13–27 weeks; and (3) third trimester, 28 weeks and above. Spontaneous abortion related to surgery was defined as an abortion occurring within 2 weeks after surgery. Leukocytosis was defined as a white blood cell count > 11,000 cells/ml.

The data analysis was carried out using SPSS 21.0 statistical analysis software (IBM Inc., Chicago, IL). Continuous variables were expressed with median and range. Continuous data were compared between groups using the Mann-Whitney nonparametric test. Statistical significance was indicated at *p* < 0 .05.

## Results

A total of 82 pregnant women who were diagnosed with AT by surgery were included in the study. The general clinical characteristics of the patients are shown in Table 1. The median age of the patients was 28 years (range, 18–38 years). Fifty-nine patients were nulliparous. Eighteen (22%) patients had had previous pelvic surgery. The median gestational age at onset was 11 weeks (range, 6–32 weeks). Among the overall population, 53 (64.6%) were in the first trimester. Seven (8.5%) patients became pregnant by in vitro fertilization and embryo transfer (IVF-ET). All the patients complained of acute lower abdominal pain; 50 (61%) patents had nausea and vomiting. Only 3 (3.7%) patients had fever. Laboratory studies revealed a mild elevation of the white blood cell count, and leukocytosis was found in 39 (47.6%) patients.

**Table 1:** Clinical characteristics of patients

Characteristics	Patients
Age (year), median (range)	28 (18–38)
Nulliparous, n/N (%)	59/82 (72.0%)
Previous surgery	
Cesarean section, n/N (%)	9/82 (11.0%)
Cystectomy, n/N (%)	6/82 (7.3%)
Appendectomy, n/N (%)	3/82 (3.7%)
Conception by IVF-ET, n/N (%)	7/82 (8.5%)
Gestational age (week), median (range)	11/82 (6–32)
First trimester, n/N (%)	53/82 (64.6%)
Second trimester, n/N (%)	21/82 (25.6%)
Third trimester, n/N (%)	8/82 (9.8%)
Symptoms	
Acute or subacute pelvic pain, n/N (%)	82/82 (100%)
Abdominal tenderness, n/N (%)	29/82 (35.4%)
Nausea and/or vomiting, n/N (%)	50/82 (61%)
Fever, n/N (%)	3/82 (3.7%)
White blood cell count(10^9^cell/l), median (range)	11.1 (6.0–20.0)

*IVF-ET* In vitro fertilization and embryo transfer

Ultrasound examination was performed for all patients. The ultrasound characteristics of the adnexal mass are shown in Table 2. In all patients except two, an adnexal or pelvic mass could be detected on emergency ultrasound. The median largest diameter of the adnexal mass was 7 cm (range, 4–14 cm). Most adnexal masses were classified as unilocular cysts (52/80; 65%), and the cyst fluid was most often described as anechoic (68/80, 85%). Doppler flow examination was performed in 48 patients, revealing a lack of flow in 30 (62.5%).

**Table 2:** Characteristics of ultrasound

Characteristics	Patients
Largest diameter of adnexal mass (cm), median (range)	7 (4–14)
Doppler signals of torsion mass	
Detected, n/N (%)	18/48 (37.5%)^a^
Undetected, n/N (%)	30/48 (62.5%)^a^
Morphological classification of adnexal mass	
Unilocular, n/N (%)	52/80^b^ (65%)
Multilocular, n/N (%)	28/80 (35%)
Echogenicity of the adnexal mass	
Anechoic, n/N (%)	68/80 (85%)
Mixed, n/N (%)	12/80 (15%)

^a^: Doppler signals were tested in 48 patients

^b^: adnexal mass was not detected in 2 patients

All patients underwent an emergency surgical procedure for their initial treatment. Laparoscopy was performed in 63 patients (76.8%). There were no conversions to laparotomy in the laparoscopy group. The median gestational age in the laparoscopy group was 10 weeks (range 6–28 weeks). The median gestational age in the laparotomy group was 26 weeks (range 8–31 weeks). The median gestational age in the laparotomy group was significantly larger than that in the laparoscopy group (*p*<0.001).

The surgical and pathological characteristics are shown in Table 3. The median interval between onset and surgery was 14 h (range, 4–192 h). Three patients received surgery because of the suspicion of appendicitis. Only the Fallopian tubes were involved in 2 (2.4%) patients. Fifty-three (64.6%) patients underwent conservative surgery, including detorsion and cystectomy in 48 (90.1%, 48/53) patients, detorsion alone in 1 patient, detorsion and cyst fenestration in 2 patients and salpingectomy only in 2 patients. Detorsion and USO were performed in 29 (35.4%) patients. The final histology was reported in 79 (96.3%) patients. The most common histopathological findings were benign, including corpus luteum cyst, serous cystadenoma, begin teratoma, follicular cyst, mesosalpinx cyst, ovarian endometrioma, mucinous cystadenoma, and ovarian clean cell carcinoma. Only 1 malignant lesion was found.

**Table 3:** Surgical and pathological characteristics

Characteristics	Patients
Duration time from onset to surgery (hours), median (range)	14 (4–192)
Surgical indication	
Suspicious of adnexal torsion, n/N (%)	79/82 (96.3%)
Suspicious of appendicitis, n/N (%)	3/82 (3.7%)
Surgical approach	
Laparoscopy, n/N (%)	63/82 (76.8%)
Laparotomy, n/N (%)	19/82 (23.2%)
Side of torsion	
Right, n/N (%)	43 /82 (52.4%)
Left, n/N (%)	39/82 (47.6%)
Organs involved in the torsion	
Ovary, n/N (%)	35/82 (42.7%)
Ovary and tube, n/N (%)	45/82 (54.9%)
Tube, n/N (%)	2/82 (2.4%)
Conservative surgery	
Detorsion only, n/N (%)	1/82 (1.2%)
Detorsion + fenestration, n/N (%)	2/82 (2.4%)
Detorsion + cystectomy, n/N (%)	48/82 (58.5%)
Salpingectomy, n/N (%)	2/82 (2.4%)
Detorsion + USO, n/N (%)	29/82 (35.4%)
Pathological results^*^	
Corpus luteum cyst, n/N (%)	35/79 (44.3%)
Serous cystadenoma, n/N (%)	14/79 (17.7%)
Begin teratoma, n/N (%)	14/79 (17.7%)
Follicular cyst, n/N (%)	8/79 (10.1%)
Mesosalpinx cyst, n/N (%)	5/79 (6.3%)
Endometrioma, n/N (%)	1/79 (1.3%)
Mucinous cystadenoma, n/N (%)	1/79 (1.3%)
Ovarian clean cell carcinoma, n/N (%)	1/79 (1.3%)

* 79 patients had available pathologic results

*USO* Unilateral salpingo-oophorectomy

All patients recovered well and had an uneventful postoperative course. No thrombotic events, sepsis or spontaneous abortion occurred after the operation. There was no recurrence of AT during the same pregnancy. The patients who underwent surgery in the first trimester received oral progesterone until 12 gestational weeks.

The obstetric outcomes of the patients are shown in Table 4. Simultaneous artificial abortion was performed in 7 (8.5%) patients, 5 in the conservative surgery group and 2 in the USO group, and the other 75 (91.5%) patients continued with their pregnancies. All 75 women had a postoperative ultrasound that confirmed the fetal pulse before discharge. One woman who underwent USO at 8 weeks of gestation experienced intrauterine fetal death at 25 weeks of gestation. Seventy-one pregnant women (94.7%) delivered healthy babies at term, and 3 (4%) delivered preterm babies at 35 to 36 weeks of gestation, with a neonatal uneventful course until discharge. The average gestational age at delivery was 38.6 ± 1.5 weeks, and the average neonatal weight was 3190 ± 282 g.

**Table 4:** Obstetric outcomes of the patients

Variable	Patients
Artificial abortion, n/N (%)	7/82 (8.5%)
Intrauterine fetal death, n/N (%)	1/75 (1.3%)
Preterm delivery, n/N (%)	3/75 (4%)
Term delivery, n/N (%)	71/75 (94.7%)
Vaginal delivery, n/N (%)	53/74 (71.6%)
Cesarean delivery, n/N (%)	21/74 (28.4%)
Gestational age at delivery (week), mean ± SD	38.6 ± 1.5
Neonatal weight(g), mean ± SD	3190 ± 282

## Discussion

In the present study, we summarized the characteristics, treatment and outcomes of AT in pregnant women over 10 years. Eighty-two patients were included. However, considering that the occurrence of AT during pregnancy is uncommon, the number of cases in our study is relatively large. Hasson [4] et al. described a series of 118 patients with AT during a 10-year period in two tertiary centers, of which 41 patients were pregnant. Ginath [14] et al. reported 54 cases of AT in pregnant women.

The accurate diagnosis of AT is often challenging, as the symptoms and signs of AT during pregnancy lack specificity. Consistent with other studies [13, 14], most cases occurred in the first trimester but could occur even in the third trimester: 8 (9.8%) patients experienced AT in the third trimester. Almost all patients were admitted to the emergency department because of acute or subacute low abdominal pain [7]. Nausea and vomiting was another common manifestation after pain, with an incidence of up to 70% [2, 4, 5, 15]. AT can be misdiagnosed as other diseases that cause lower abdominal pain, such as appendicitis and renal colic [15]. In the present study, three patients underwent laparotomy because of suspected appendicitis and were found to have AT during the operation. In addition, white blood cell counts may be slightly elevated. Chang [15] et al. reported that 45% of patients had elevated white blood cells. Ginath [14] et al. also revealed a slight elevation in white blood cell counts in pregnant women with AT compared with nonpregnant women. In our study, 47.6% of patients had leukocytosis, which was consistent with previous studies.

Ultrasound is the most common imaging method for evaluating acute abdominal pain during pregnancy in the emergency department. Color Doppler sonography has been recently proposed as a useful tool for improving the preoperative diagnosis of AT. However, some studies have demonstrated that the Doppler finding has a high false-negative rate. Smorgick [3] et al. reported that a normal Doppler flow was found in 60% of cases of AT in pregnant women. Hasson [4] et al. described that only 39% of cases of AT in pregnant women showed no blood flow on the Doppler test. Ginath [14] et al. reported that Doppler flow examination revealed the lack of arterial flow in 70% of pregnant women. In the present study, 37.5% of the patients had a normal Doppler flow signal. The reason for the high false-negative rate may be that the severity of the vascular impairment is variable, depending on the number of twists, the tightness and the duration of torsion, which can cause partial or complete vascular obstruction. Therefore, the decision regarding surgery should not be made based on the findings regarding blood flow alone but based on the clinical suspicion of AT. In addition, the gravid uterus may displace the twisted mass so that the mass is not detected, which can lead to delayed diagnosis. In our study, ultrasound failed to reveal the twisted mass in 2 patients due to the enlarged uterus. There is increasing evidence that MRI can be used for the primary evaluation of acute abdominal pain in pregnancy, particularly when appendicitis cannot be excluded and a mass is not detected by ultrasound [16].

Similar to in nonpregnant women, laparoscopy has become a common and safe mode of surgical treatment for AT in pregnant women. Hasson [4] et al. reported that laparoscopy was performed in 88% of pregnant women with AT. Daykan [6] et al. conducted a study in which 85 pregnant women with AT were enrolled, and 78 (91.7%) patients underwent laparoscopy. In 2011, the Society of American Gastrointestinal and Endoscopic Surgeons stated that laparoscopy can be safely performed during any trimester of pregnancy and is recommended for the diagnosis and treatment of AT unless the clinical severity warrants laparotomy [17]. Laparoscopy can be performed in the third trimester by experienced surgeons. Chohan [18] successfully performed laparoscopic surgery for a woman with fallopian tube torsion at 35 weeks of gestation. It has been confirmed that laparoscopic surgery does not affect obstetrical outcomes compared with laparotomy [15] and does not increase complications such as thromboembolism events, sepsis and spontaneous abortion.

Compared with nonpregnant women with AT, pregnant women were more likely to undergo conservative surgical management. Previous studies have shown that 30% ~ 100% of patients underwent conservative surgical management, and detorsion only was the most common procedure [3, 4, 7, 14]. But in our study, only one patient underwent detorsion only and 48 underwent cystectomy. Interestingly, there was no recurrence in our study during the same pregnancy. This is inconsistent with previously published literatures. Hasson [4] et al. reported that the rate of recurrence in the same pregnancy of AT for pregnant women who underwent detorsion only was 19.5%. Pansky [19] et al. described that the recurrence rate of detorsion only was 20% and that there was no recurrence in patients who underwent cystectomy or oophorectomy. Compared with the previous studies, cystectomy may remove the risk factors for recurrence. The twisted adnexal mass has edema and is fragile, which sometimes makes cystectomy difficult. In this situation, detorsion and fenestration for large cysts can be performed safely because most of the cases are benign masses.

Regrettably, more patients (35.5%, 29/82) in our study underwent USO than in previous studies [4, 6, 12, 14]. The decision to perform USO was at the discretion of the surgeon. Based on the descriptions in the surgical records in the present study, physicians preferred to perform USO if the color of the adnexa was still blue-black for ten minutes after detorsion. However, some studies have proven that it is not accurate to determine the activity of the ovary based on the color during the surgery [20–22]. Parelkar [22] et al. presented a case series of 12 children with AT. Although the appearance of the ovaries of 10 patients was found to be severely ischemic during the operation, all underwent detorsion with or without evacuation of the hematoma. Follow-up sonography showed all ovaries had follicular development except one.

Surgical procedures did not affect the obstetric outcomes. In our study, there was no spontaneous abortion after surgery. One patient experienced intrauterine fetal death at 25 weeks of gestation. Wherever the patient underwent oophorectomy at 8 weeks of gestation, fetal loss was not associated with the surgery. Inconsistent with our observation, in a study of the same size, Daykan [6] et al. reported that the overall postoperative spontaneous abortion rate was 3.5%, but all occurred more than 2 weeks after the surgery, so they were not considered to be related to the surgery. Before the formation of the placenta, removing the ovarian or corpus luteum may decrease the level of progesterone, which maintains the development of the fetus. According to the guidelines of the Society of American Gastrointestinal and Endoscopic Surgeons [17], progesterone was administered to patients who underwent surgery in the first trimester in our study. This may be the reason for the relatively low rate of spontaneous abortion after surgery.

However, our study also has weaknesses, namely, its retrospective nature and long-time span. The limited sample size and bias caused by a single center analysis may have also affected the results of the study. However, the low incidence rate makes it difficult to collect enough cases to conduct a prospective study.

## Conclusions

AT is a rare complication of pregnancy. Acute pelvic pain, pelvic mass and nausea and vomiting are the main symptoms. Doppler flow examination has a high false-negative rate. The decision regarding surgery should be made as soon as possible according to the clinical suspicion of AT. Laparoscopy is a safe approach to diagnose and treat AT. Conservative surgery, including detorsion with cystectomy or fenestration, can not only preserve ovarian function but also decrease the recurrence rate.

## Data Availability

The datasets used and/or analysed during the current study are available from the corresponding author on reasonable request.

## Abbreviations

AT: Adnexal torsion
USO: Unilateral salpingo-oophorectomy
